# Vibrotactile Feedback for Brain-Computer Interface Operation

**DOI:** 10.1155/2007/48937

**Published:** 2007-09-03

**Authors:** Febo Cincotti, Laura Kauhanen, Fabio Aloise, Tapio Palomäki, Nicholas Caporusso, Pasi Jylänki, Donatella Mattia, Fabio Babiloni, Gerolf Vanacker, Marnix Nuttin, Maria Grazia Marciani, José del R. Millán

**Affiliations:** ^1^Laboratory of Neuroelectrical Imaging and Brain Computer Interface, Fondazione Santa Lucia IRCCS, Via Ardeatina 306, 00179 Roma, Italy; ^2^Laboratory of Computational Engineering, Helsinki University of Technology, P.O. Box 9203, 02015 TKK, Finland; ^3^Department of Computer Science, University of Bari, Via Orabona 4, 70125 Bari, Italy; ^4^Dipartimento di Fisiologia Umana e Farmacologia, Università degli Studi di Roma “La Sapienza”, Piazzale Aldo Moro 5, 00185 Roma, Italy; ^5^Department of Mechanical Engineering, Katholieke Universiteit Leuven, Celestijnenlaan 300 B, 3000 Leuven, Belgium; ^6^Dipartimento di Neuroscienze, Università degli Studi di Roma “Tor Vergata”, Via Montpellier 1, 00173 Roma, Italy; ^7^IDIAP Research Institute, 1920 Martigny, Switzerland; ^8^Ecole Polytechnique Fédérale de Lausanne (EPFL), 1015 Lausanne, Switzerland

## Abstract

To be correctly mastered, brain-computer interfaces (BCIs) need an uninterrupted flow of feedback to the user. This feedback is usually delivered through the visual channel. Our aim was to explore the benefits of vibrotactile feedback during users' training and control of EEG-based BCI applications. A protocol for delivering vibrotactile feedback, including specific hardware and software arrangements, was specified. In three studies with 33 subjects (including 3 with spinal cord injury), we compared vibrotactile and visual feedback, addressing: (I) the feasibility of subjects' training to master their EEG rhythms using tactile feedback; (II) the compatibility of this form of feedback in presence of a visual distracter; (III) the performance in presence of a complex visual task on the same (visual) or different (tactile) sensory channel. The stimulation protocol we developed supports a general usage of the tactors; preliminary experimentations. All studies indicated that the vibrotactile channel can function as a valuable feedback modality with reliability comparable to the classical visual feedback. Advantages of using a vibrotactile feedback emerged when the visual channel was highly loaded by a complex task. In all experiments, vibrotactile feedback felt, after some training, more natural for both controls and SCI users.

## 1. INTRODUCTION

The human brain relies on inputs from different senses to form percepts of objects and events, during everyday life. These pieces of information usually complement and confirm each other, thereby enhancing the reliability of percept [1]. Somatosensory feedback is a vital component of motor planning, control, and adaptation, and there is a growing effort to include this feedback modality in neural prosthetic systems [2].

Visual presentation of stimuli is the most common feedback modality in neurofeedback paradigms for self-regulation of the brain's electrical activity. Thus, it is comprehensible that current brain-computer communication systems mainly operate with visual stimuli [3]. However, components of the visual system such as vision, visual attention, and focusing gaze are physiologically engaged during the dynamic contact between the body and environment. Furthermore, the visual sense may be compromised in some patients who are in need of BCI support. Thus, towards more efficient brain-computer communication, it seems important to also obtain evidence of how the extravision somatosensory modality performs during self-regulation of the brain's electrical activity.

Only few studies have tested other feedback modalities for brain-computer interfaces (BCIs). Hinterberger [4] and Pham [5, 6] tested auditory feedback, but, to our knowledge, no one has trained subjects with tactile feedback. Vibrotactile stimuli have been previously used [7, 8] for BCI operation in a different context, that is, as an external driving stimulus to elicit exogenous EEG rhythms.

In addition to freeing visual and auditory attention, tactile stimuli are more natural in a manipulation task than, for example, visual stimuli. Even though BCI training is strongly dependent on feedback, surprisingly, only two studies have explored how feedback affects the learning process. McFarland [9] investigated what happens when feedback is removed from well-trained subjects and Neuper [10] compared continuous and discrete feedback. No guidelines exist regarding somatosensory stimulation for BCIs.

This study aims to explore the benefits of vibrotactile feedback for user training and accurate control of an EEG-based brain-computer interface.

## 2. VIBROTACTILE STIMULATION

### 2.1. Physiological perception

Several receptors for the transduction of mechanical solicitation on the skin into neuronal signals are available in man: Merkel's receptors, which are slow adapting receptors with high spatial resolution; the Meissner's corpuscles, present in the glabrous skin (lips, finger), with characteristic of rapid adaptation and high spatial resolution; the Pacini's corpuscles which detect very rapid vibration and are quickly adapting. The somesthetic information travels from the receptors to the central nervous system using the fastest communication lines in the human body; the so-called dorsal-lateral column way, delivering information at a speed of over 100 m/s. This somesthetic system delivers very precise information of which two neighboring points on the human skin can be perceived as distinct. The spatial resolution of the skin has been tested since 1826 [11] using static pressure stimuli showing that it varies along the body, ranging from few millimeters (fingers) to more than four centimeters (trunk).

Vibrotactile devices delivering variable pressure on the skin have been employed as an alternative sensitive channel for blind or deaf individuals [12, 13]. The sensitivity for vibrotactile stimulation depends on body position and age of the subjects [14]. Frequency of vibration is a second parameter that influences the quality and intensity of perception, being modulated by factors like body position, skin temperature, and underlying tissue (bone, fat, muscle, or a combination). Values between 50 and 300 Hz should generally be chosen. The use of oscillating pressure also adds new degrees of freedom to the design of vibrotactile stimuli, such as waveform shape, for example, sinusoidal or square and amplitude modulations (at different modulation frequencies) of the carrier frequency.

In summary, several features of vibrotactile stimuli can be modulated to convey information over this sensory channel. The list can be divided into two subsets. The first includes features related to physical perception:

1. *frequency*, the main spectral component of the periodic stimulus;
2. *intensity*, the strength of stimulation (measured either as force applied or as displacement produced);
3. *timbre*, the complexity of the stimulation waveform (i.e., the content of harmonics in the spectral representation);
4. *duration*, the time length of the “on” time or an elementary stimulation;
5. *spatial location*, the single body part or the pattern of parts that are stimulated.

Features in the second subset are clearly perceived by an individual, but do not rely on any property of the receptors (e.g., need to be interpreted on a cognitive level):

1. *rhythm* , the sequences of stimulation and pauses, with specific durations, that compose the current message, that is, a triplet of stimuli, a Morse coded SOS, and so forth;
2. *tempo* , the fastness, due to longer or shorter duration of the whole message, given at fixed rhythm;
3. *flutter* , an amplitude modulation of the stimulation carrier frequency that can either be perceived as increase and decrease of the intensity (if modulation is slower than 5 Hz) or as “roughness” (if modulation is faster than 10 Hz).

Given the hardware at our disposal (described below), we designed an appropriate software framework to test the effectiveness of all the features mentioned above, as to maximize the capacity of the vibrotactile channel.

### 2.2. Generation and delivery of stimuli

**Figure 1:**
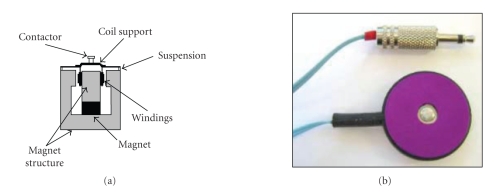
(a) Components of a C-2 tactor; (b) External aspect.

We used C-2 tactors (Engineering Acoustics, Inc, Winter Park, FL, USA) (see Figure 1) that are magnetic actuators, similar in principle to audio speakers; the current flowing in their coil pushes a central structure named contactor against the skin and back. Different from acoustic transducers, the structure is tuned on a narrow band around 250 Hz, so only signals at these frequencies can be effectively transduced.

By driving a tactor with two mixed sine waves, complex waveforms can be obtained. Moreover, a third auxiliary input can be fed at the amplification stage. Even if efficiency issues suggest not to deviate from the resonance frequency of 250 Hz, the frequency of stimulation can be selected by the user. The output intensity can be set to four different values (amplification gains). A peripheral interface controller (PIC) included on the control board takes care of serial communication with the PC, and sets in the generation and amplification subsystems the appropriate values of frequency and gain. By using a battery as power supply and a serial port to a Bluetooth (BT) adapter, the host PC can send commands over the air to the vibrotactile device. Since both the control board and the BT adapter are battery powered, the users can wear a wireless system during the experiments.

**Figure 2:**
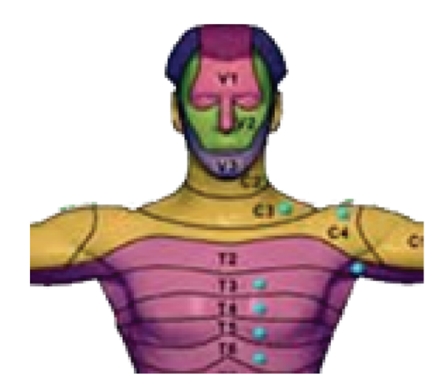
Dermatomes of the human body, that is, the levels innervated by each pair of spinal roots. A spinal cord injury may leave denervated all dermatomes, below the level of the lesion.

The tactors are relatively lightweight and small (∼4 cm diameter). The skin contactor is 7.5 mm diameter, raised to 0.6 mm from the surface of the case, so that it can be pre-loaded on skin. Since the nominal displacement is about 0.6 mm, the tactor-skin adhesion is never lost in any phase of the vibration. In principle, taping them to the body could be a solution for short term experimentations, but it is hardly satisfactory for longer sessions. The ideal placement technique should (i) be easy to wear, (ii) be comfortable to wear, (iii) guarantee good adhesion to skin, (iv) allow good skin sensitivity. Moreover, we need to take into account the possibility that some motor-disabled users could be spinal cord injured (SCI) suffering from sensory deficits in the lower part of their body. The position of tactors must be above the dermatome corresponding to the level of the lesion (see Figure 2).

**Figure 3:**
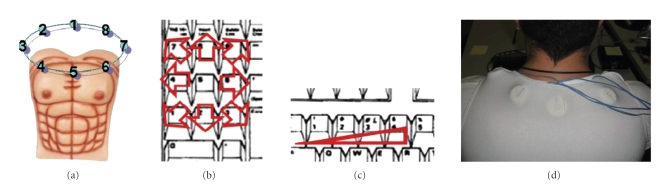
(a) Tactors indexing, used in the results. (b) Mapping of perceived position of stimulation to keys of a computer's numeric keypad; correspondence does not rely on numbers, but on directions (e.g., leftmost key coded as a perceived stimulus to the left). (c) Mapping of perceived intensity of stimulation to keys of a computer's keyboard; 1 coded the weakest intensity, and 4 the strongest. (d) Montage of tactors on the subject's body.

We defined a common standard placement of the tactors where they are placed on the shoulders of the users, using an elastane T-shirt to keep them steadily in position and slightly pressed against the skin (see Figure 3(d)).

The software framework designed to drive the vibrotactile actuators was divided into several layers, aiming at implementing commands at different levels of abstraction. In particular, primitive stimulation modalities (tactons, [15, 16]) were used as a general library of stimuli.

## 3. PRELIMINARY EXPERIMENTATIONS

In the preliminary experiments, our aim was to address whether a subject is able to distinguish two separate stimuli delivered through the experimental setup described above, even when the stimulation characteristics are only slightly different. The vibrotactile features under investigation in this experiment were intensity and position.

### 3.1. Experimental setup

Five able bodied subjects (AB, one female) and three subjects with spinal cord injuries —leading to paraplegia (SCI, lesions from T3 to T8, all male) —, (SD) years old, were enrolled.

Eight tactors were positioned in a circle at even angles on the upper part of the trunk of the subjects (see Figure 3). The tactors were placed over a T-shirt, and kept in place by a second elastic T-shirt, which also provided the necessary pre-load. To avoid slipping and to help the preload even where the elastic force of the T-shirt is low, for example, between the scapulae, a circular sponge of appropriate size was stuck to the back side of each tactor.

Vibrotactile stimuli were given at 250 Hz, lasting for 500 milliseconds. During the tests, 256 separate stimuli were delivered to each subject, in four runs separated by short breaks. Each stimulus could be given to one of the eight tactors, and could have an intensity level of one to four. Positions and amplitudes were pseudorandomized, making sure that the total number of stimuli of each type would be the same.

In response to each stimulus, the subject had to respond with the perceived direction and intensity. A computer keyboard was used for this purpose. As shown in Figure 3, eight keys on the numeric keypad (pressed with fingers of the right hand) coded the perceived position, while four keys of the left part of the keyboard (pressed with fingers of the left hand) coded the perceived intensity. Two additional keys were provided for the subject to express his/her inability to recognize position or amplitude of the stimulus.

Before recording the response, the subjects practiced for a few minutes, until they declared to feel familiar with the stimuli and with the response procedure. During the practice period, the experimenter informed the subjects about the actual stimulus delivered, so that subjects could learn the association between stimulus and perception.

### 3.2. Results

During the practice session, all subjects reported that they could perceive the stimulation, even at the lowest intensity. Some subjects reported discomfort with tactors number 1 and 5, since they stimulated a region of the body where a bone (sternum or spine) was immediately below the skin. The average response time of the two key presses, direction, and amplitude was 2.35 ± 0.52 seconds.

**Figure 4:**
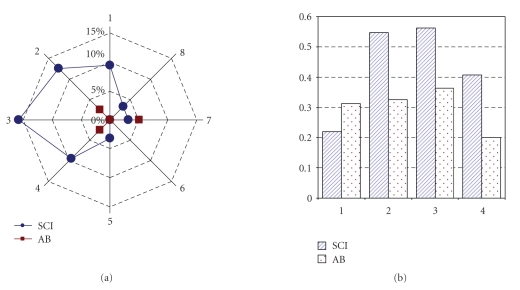
Error occurrence, divided for position (a) and for intensity (b) of stimulation. SCI and AB groups are plotted separately.

Overall errors in detecting the correct position were 3.8% and errors in detecting the intensity of stimulus were 35.9%. In both conditions, 0.2% of the responses were not classified. The distribution of errors as a function of positions and intensities is shown in Figure 4 for the SCI and the AB group separately. Most of the errors for the SCI group were made with stimuli delivered to the right part of the body; intermediate intensities were difficult to recognize for both groups.

**Figure 5:**
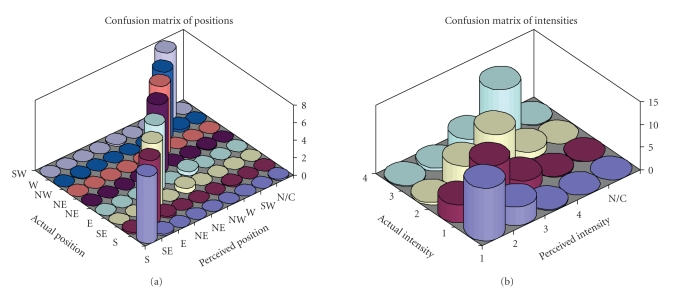
Graphical representation of confusion matrices of classification errors, that is, the number of stimli delivered to an “actual position” that were perceived as delivered to a “perceived position.” Figures represent the grand average over all subjects participating in the experiment. Ideally, all nonzero values should lie on the main diagonal.

Figure 5 shows the grand average confusion matrices of errors as a function of positions and intensities over all subjects; errors in detecting positions are almost exclusively confined to neighboring position. Errors in amplitude detection are more frequent, but mostly confined to adjacent (slightly higher or lower) intensities.

### 3.3. Discussion

Both subject groups could reliably distinguish stimuli delivered to the 8 tactors, with acceptable classification error. Errors were higher, but acceptable, on SCI subjects, possibly due to specific loss of sensitivity. A neurological examination and a preliminary experimentation to detect an individual optimal montage should be considered for further experimentations. All SCI subjects included in this study had lesions to toracic vertebrae. Lesions at higher level may prevent this solution to be effective. In such a case, an experiment in which analogous stimuli are delivered in different sites of the neck should be carried out, to assess an appropriate solution.

The pattern of direction errors shown in Figure 4 has no apparent physiological explanation. Since most of the errors are contributed by SCI subject, they should be discussed on an individual basis, using the result of the neurological examination. Possibly, a longer and more structured subject training period could help reduce misclassifications.

Intensity seems to be more difficult to classify, at least with the level of discrimination used in this experiment (4 levels). Errors mostly occur for the intermediate levels (2 and 3). Levels 1 and 4 seem to be less affected, possibly because they can be confused with only one neighboring intensity. Reducing the number of levels to two could bring misclassifications to an acceptable value.

Even though adaptation was not explicitely explored in this study, none of the subjects reported a reduction of the sensorial response with time. This was possibly prevented by the type of vibrotactile stimulation, which was not continuous, but intermittently on and off, as ruled by the temporal and spatial pattern of stimulation.

During this study, we did not experience artifacts on the EEG recordings produced by activation of the vibrotactile transducers.

Finally, due to discomfort of tactors placed in a bony region of the body (above the sternum and the spine) reported by some subjects, a slight rotation of the tactor configuration is suggested.

## 4. EXPERIMENTAL STUDIES

From the considerations of physiological and technical natures expressed so far, it is evident that somatosensory feedback is a vital component of motor planning, control, and adaptation, and there is a technical possibility to include this feedback in neural prosthetic system. To achieve this goal, it is first necessary to assess how the feedback channels would affect the training processes and compare them to the use of the dominant visual channel.

To this end, we aim to answer the following questions.

1. Can the vibrotactile channel represent valuable feedback by conveying information of subject performance during BCI training, especially compared to the classical visual channel?
2. Could vibrotactile feedback effectively integrate (or complement) visual feedback when the visual channel is engaged in monitoring the task, which is being controled using commands issued through a BCI?

In a *first experiment* , untrained subjects were trained to control a two-class BCI while receiving either visual or vibrotactile feedback. The subject performance achieved under both feedback modalities was compared (in terms of performance accuracy). Care was taken regarding the subject “adaptation” to the feedback modality by randomizing the delivery of visual and vibrotactile stimului.

In a *second experiment* , untrained subjects were exposed to both visual and/or vibrotactile feedbacks, which informed subjects about the classification outcome of a two-class BCI. We implemented an experiment in which a robot simulator program was included to mimic a distracting environment element (engaging the visual channel). This experiment addresses the question of using visual attention for monitoring the robot performance rather than the BCI performance.

Along with the previous experiments, where untrained subjects are gradually exposed to different feedback modalities with an element of “distraction,” in a *last experiment* we mimic a “real-life” condition wherein subjects are engaged in a complex visual task (which requires focused visual attention) and simultaneously they receive the necessary continuous information about the status of the system they are using.^1^ BCI trained subjects were thus exposed either to a visuovisual or to a visuovibrotactile feedback of the outcome of BCI control and overall task, respectively, to assess whether the vibrotactile information may effectively complement the visual channel.

### 4.1. Study I

In the first study, we compared visual and vibrotactile feedbacks in a short experiment (six 7-minute sessions). When imagining left- and right-hand movements, six novice subjects received either visual (three sessions) or vibrotactile (three sessions) feedback of the classification performance of the BCI. Using correct class information, the classifier was updated after each prediction. Thus, up-to-date feedback could be given throughout the experiment. Model parameters were trained online and no separate offline training session was needed.

#### 4.1.1. Material and methods

*Subjects*. Six right-handed subjects (20–30 years), who had no previous experience of BCIs, participated in the experiment.

*Recordings*. EEG was measured in a shielded room at 12 locations over the sensorimotor cortices. Signals from only two channels, C3 and C4, were used for BCI control. The sampling frequency was 500 Hz and the reference was situated between Cz and Fz.

*Experimental setup*. During the whole experiment, subjects were shown a visual target either on the right, left, or upper side of a small display in the middle of the screen. The subjects imagined either left- or right-hand movements, or did nothing (target up). The target was changed randomly every 10–15 seconds. The experiment was divided into six 7-minute sessions. Small breaks were kept between sessions. S1–S3 received vibrotactile feedback in the first three sessions and visual feedback in the following three sessions. The order was reversed for S4–S6.

*Features*. Movement-related activity (7–13 Hz) was used. FFT components were calculated from a 1 seconds time window, resulting in 2 channels × 7 frequencies = 14 features. The window was moved and features were recalculated once the classifier function had finished with the previous sample (∼every 100 microseconds).

*Classification*. A linear model with logistic output function was used to classify the features. The model was re-trained after each new feature sample (∼every 100 microseconds) using a maximum of 300 previous labeled samples (∼30 seconds) from both classes (less in the beginning of the experiment). The iterative least squares algorithm was used to update the model parameters. Classification and training was done only when the subject was performing either the left or right task.

*Feedback*. Vibrotactile feedback vibrating at 200 Hz and lasting for ∼100 microseconds was delivered either to the left or the right lower neck through the vibrotactile transducer. The amplitude was set to a value that the subjects reported being clearly perceivable. Visual feedback showed for ∼100 microseconds an arrow on the screen either to the left or right. Feedback was given once every second if the averaged posterior probabilities of 10 previous predictions exceeded 70% (S1 and S4) or 60% (others) for either of the two classes, that is, feedback was not given in uncertain cases. Feedback was given from the beginning of the experiment. No feedback was given during the target-up case.

#### 4.1.2. Results

Table 1 shows the mean classification accuracy averaged over three sessions with different feedback modalities. Even during the short 42-minute experiment, high-classification accuracies (means 56–80') were possible in some subjects.

Contralateral slow somatosensory evoked potential (SEP) could be detected in all subjects at 200 microseconds. The small visual feedback does not evoke any clear response. The vibrotactile feedback does not, however, show significant difference in the alpha-band frequencies that could interfere with the classification of motor imagination.

#### 4.1.3. Discussion

No differences were found between training with vibrotactile or visual feedback during the 42-minute experiment. This indicates that, vibrotactile feedback could be used as an alternative to visual feedback if, for example, visual attention is needed for other tasks. These results should, however, be verified with more subjects. When asked, most subjects thought vibrotactile feedback felt more natural. However, one subject said that it sometimes, especially during misclassifications, interfered with the imagination of movements. Feedback was given discretely because continuous vibrotactile feedback was not possible due to technical difficulties. Even though SEPs can be detected in the averaged signals, the vibrotactile feedback did not interfere with the classified brain signals in the 7–13 Hz range.

### 4.2. Study II

Study II continues the work of study I by comparing visual and vibrotactile feedback in a short experiment (nine 4.5-minute sessions). As in study I, six novice subjects received feedback of the classification performance of the BCI when imaging left- and right-hand movements. The experimental paradigm of study I was, however, slightly changed.

First, we used no threshold when giving feedback and thus feedback was always given once a second. Second, we used instant band power values and more several channels as features; these more sophisticated features require a feature selection, which we did during the first three breaks. To ensure that the reselection of features did not interfere with learning, we used the same features in the last six sessions. Third, in addition to feedback, a robot simulator program was also shown during the whole experiment on the screen to mimic a distracting environment of a BCI that generates a high visual workload.

#### 4.2.1. Material and methods

*Subjects*. Six right-handed subjects (22–26 years, one female) with no previous experience of BCIs participated in the experiment.

*Recordings*. EEG was measured at 13 locations over the sensorimotor cortices (Fz, Cz, Pz, FC1, FC2, CP1, CP2, C3, C4, FC5, FC6, CP5, CP6) with a Brain Products 32-channel active electrodes system. The electrodes on the cap had built-in amplifiers, impedance level indicators, and active electromagnetic shielding. The sampling frequency was 500 Hz and the reference electrode was situated between Fz and Cz.

**Figure 6:**
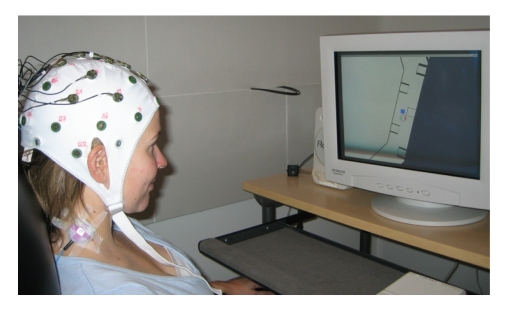
Subject seated in front of screen. Both visual and vibrotactile feedbacks are given.

**Figure 7:**
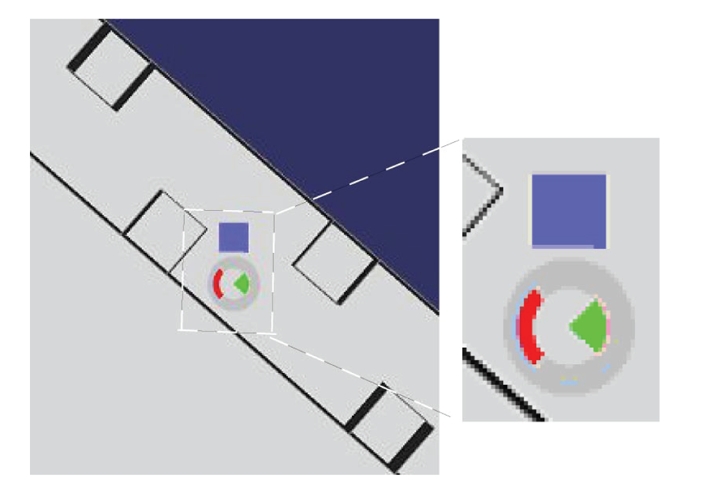
Top view of robot simulator program. The red task indicator showing left movement and visual feedback (green) showing feedback to the right side are displayed below the blue robot.

*Experimental setup*. Subjects were seated comfortably in a shielded room, in front of a monitor that displayed the top view of a simulated wheelchair (see Figure 6). The wheelchair was autonomously controlled by the computer; the purpose of the environment was to distract the subject by generating a high visual workload while making the change of target predictable. The target task indicator was situated below the wheelchair (see Figure 7). The wheelchair environment was an infinite circular corridor with obstacles requiring left and right turns.

The task indicator displayed a red target in either left, right, or up position. Subject's task was to imagine kinesthetic left- and right-hand movements or to do nothing (target up). The targets were predictably changed online by the experimenter from left target to right target (going through up target) and in reverse order. This suited the path of the robot best and made it easier for the subjects to prepare for the upcoming movement. Each left and right task lasted 5–10 seconds and each up-task lasted 1–2 seconds. The left and right tasks were alternated to approximately match the path taken in the environment by the wheelchair.

The experiment consisted of nine 4.5 minute sessions. In the first session, there was no feedback. In the next two sessions, both vibrotactile and visual feedbacks were presented simultaneously to familiarize the subject with them. Subjects S1–S3 received vibrotactile feedback in the next three sessions and visual feedback in the last three sessions. For subjects S4–S6, in the last six sessions, the order of the feedback modalities was changed.

*Features*. For each channel, one instant spectral/band power value was used as feature; the features were calculated once every second by convolving the EEG signals with Gabor filters. The length of each Gabor filter was two seconds corresponding to a bandwidth of approximately 0.5 Hz. The center frequency of each filter was determined in the feature selection from the 6–30 Hz frequency band.

*Feature selection*. Subject-specific center frequencies, as well as the classification relevance of each channel, were determined using Bayesian inference. Markov chain Monte Carlo (MCMC) methods were used to draw samples from the joint posterior distribution of the model parameters and input features. Reversible jump Markov Chain Monte Carlo (RJMCMC) was used to jump between models with different input feature combinations [17]. Joint probability of each channel and frequency component was determined based on the number of “visits” during the sampling process. As a criterion for selecting features, we required a given channel and the corresponding centre frequency to be included in the model with sufficiently high posterior probability; we chose six or more of the most probable features for which the joint probability exceeded 0.25.

Feature selection was done during the breaks after sessions 1, 2, and 3. After sessions 1 and 2, the best Gabor frequency for each channel was determined using the data from the previous session. This Gabor frequency from all 13 channels was included in the model used in the following sessions, 2 and 3 correspondingly. After the third session, using data from sessions 2 and 3, RJMCMC sampling of input channels was combined with the sampling of the Gabor frequencies to determine the final channels and Gabor filters as features.

*Classification*. The classifier parameters were updated online once every second with the iterative least squares algorithm [18]. A prediction of the current class was made once every second for the newest sample before retraining of the model; a maximum of 300 most recent samples (5 minutes of data) with correct class labels was used as training data for each class.

In sessions 2–5 and 7-8, the model was trained online once every second. In the beginning of sessions 2 and 3, a model was used that was trained with the selected features using the data from the previous session. During the third break, the final features were used to initialize a model which was then used in sessions 4 and 7; the resulting models were then continued to be trained in sessions 5 and 8. The obtained models were tested, without training them, in sessions six and nine.

*Feedback*. The subject was given visual and/or vibrotactile feedback once every second. The visual feedback was displayed as a rose in the middle of the simulator screen with a green segment to each of the four directions. The left and the right segments were lit for 200 microseconds corresponding to the output of the classifier (see Figure 7). The vibrotactile feedback was given for 200 microseconds at 200 Hz with vibrating elements attached with tape to the subject's left- and right-side of the lower neck.

#### 4.2.2. Results

Five subjects achieved high overall classification accuracies, on average as good as 59–79%, in the vibrotactile (HF), and visual feedback (VF) sessions (see Table 2). The subjects performed between 160 and 247 trials per session. S6 did not gain control over chance level (50–54%). S1 obtained an average accuracy of 79% for both feedback modalities and S4 reached 79% for the visual feedback modality. The average accuracies in the training sessions (TS) were 6-7% lower than the average accuracies in HF and VF sessions. We found no differences between average accuracies of the VF and HF sessions.

A response to the vibrotactile stimulation appears in the 0–8 Hz and 30–40 Hz bands in synchrony with the onset and end of the vibrotactile stimulation. In the event-related potentials (ERP) to the vibrotactile stimulation, low-pass filtered below 10 Hz, an N200 peak can be seen in both hemispheres during left- and right-side vibrotactile stimulation. However, both these responses have no role in real-time classification using time-frequency transformations in the 8–30 Hz frequency range.

#### 4.2.3. Discussion

This study confirmed the results of study I; no differences were found between training with either vibrotactile or visual feedback, during the short 41-minute experiment. These results show that vibrotactile feedback could be used as an alternative to visual feedback when starting to learn to use a BCI. The choice of feedback modality is therefore largely dependent on subjects' preferences, indented environment of use, and the application. The use of the robot simulator program as a distracter did not disturb the subjects training. As in study I, when asked, most of the subjects felt that vibrotactile feedback was more natural. S1, S2, and S4 indicated that in the case of conflicting feedback, vibrotactile feedback was more disturbing than visual feedback. Even though vibrotactile responses could be detected in the averaged signals, the vibrotactile feedback did not interfere with the classification of the brain signals in the 6–30 Hz range.

S2–S5 performed better during the first feedback sessions compared to the second ones, independently of the type of feedback. This can partly be explained by the fact that the same model was used. Because the model was initialized after session 3 it was not as much up-to-date as during the second feedback modality. The model was not updated during the testing sessions explaning why the results of the training sessions are better.

From study I and II, we conclude that short-term learning is possible also with vibrotactile feedback. New experiments with more subjects are needed to evaluate the longer-term benefits of vibrotactile feedback. It should also be tested whether vibrotactile feedback is useful to patients with reduced sensing capabilities.

### 4.3. Study III

In study III, subjects were exposed to a joint visual and vibrotactile feedback, to assess whether the vibrotactile information could complement the visual channel for feedback, when the visual channel is intensively used as a carrier of task-related information. This experimental condition mimicked a “real-life” condition where the subject, engaged in a given task (spatial navigation), could simultaneously receive continuous information of his control strategy (feedback). Continuous state of the control signal, rather than a time-discrete classification was fed back to the user. To better focus on the properties of feedback and to reduce intersubject and intersession variabilities, due to different levels of training and fatigue, the “BCI” control signal was not derived by modulation of subject's brainwaves, but simulated by the movement of a PC mouse, to which a BCI-derived noise was added.

#### 4.3.1. Material and methods

**Figure 8:**
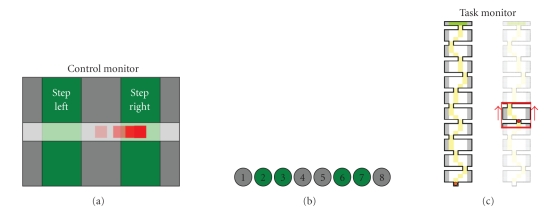
*Panel* (a): visual feedback of the pseudo-BCI controller; the subject had partial control on the red cursor, whose position was converted at discrete times (2 seconds) into navigation commands (step left, right, or no stepping). *Panel* (b): vibrotactile feedback of the pseudo-BCI controller; each tactor of the stripe encoded the tactile version of the visual cursor. *Panel* (c): scheme of the task; the drawing to the left represents the whole maze, with the ideal path marked in yellow. In the drawing to the right, the scrolling red frame shows the portion of the maze visible at once of the task display.

Thirteen subjects, two of which suffered from paraplegia due to lesions to their spinal cord, were involved in the experimentation. The experimental task consisted of moving a placeholder visible on a “task” monitor, with the goal of stepping through a sequence of 10 “rooms” (see Figure 8(c)), following a path constrained by narrow “gates” between adjacent rooms.

**Control monitor**

Subject's intention to move the placeholder was mediated by a BCI-like controller. In a first setting, the visual feedback of this controller was visible in a “control monitor” (see Figure 8(a)). The horizontal position of a cursor was partially regulated by the subject, moving a computer mouse. In fact, the cursor movement was affected by noise and delay, so that (inaccurate) motion was as similar as possible to a typical BCI-controlled cursor trajectory. To achieve this goal, the processing chain of the BCI2000 software [19] was set up like in a mu rhythm-based cursor control task, except that the amplitude of the spectral “EEG” component of interest was modulated by the mouse position. In addition, the time series of cursor drifted from an actual EEG-modulation recording was added sample by sample to the cursor control signal.

**Figure 9:**
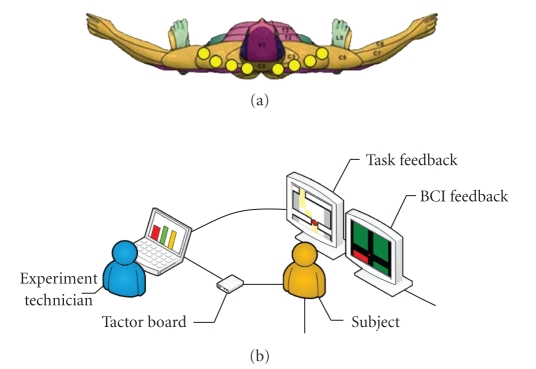
*Panel* (a): positions of the stripe of tactors on the subject's shoulders. *Panel* (b): experimental setup for visual feedback; the monitors in front of the subjects show the navigation task (task monitor, top) and the pseudo-BCI feedback (control monitor, bottom).

In a second setting, the feedback of this BCI-like controller was given through a stripe of eight tactors (see Figure 8(b)), positioned on the shoulders of the subject as shown in Figure 9(a). Only one tactor at a time was active, encoding information about the horizontal position of a tactile cursor.

Once every 2 seconds, the (visual or tactile) cursor's horizontal position was sampled and compared to the limits of the five intervals defined on the screen; and the placeholder moved one step to the right, to the left, or stayed in its line, accordingly (see Figures 8(a) and 8(b)). If not impeded by a transverse “wall,” the placeholder moved one step ahead at each time. Since the extreme left and right position of the control cursor did not produce a lateral movement of the placeholder, the subject could not simply grossly move the cursor in one direction, but had to attend the visual feedback on the control monitor, to make sure he did not under- or over-shoot cursor's position (which would be a too easy control strategy). This designed produced (i) the need of attentive level, and (ii) a number of mistakes that were comparable to real BCI operation.

Subjects practiced for 30 minutes with the control monitor alone with both visual and tactile feedbacks to stabilize performance before challenging the task.

**Task monitor**

Each room of the navigation space measured 4 × 4steps and access to the following room was allowed only through a narrow “gate.” In the task monitor, movement was strongly discretized (one step every 2 seconds), so that the subject could not infer the status of the controller by looking at the placeholder's motion.

To force subjects to keep their visual attention on the task monitor, a colored green or yellow key appeared at random times once or twice for each “room.” Before proceeding to the next “room,” the subject had to report the color of the last key. If wrong, the subject had to navigate again the same room, thus making the path to the final goal longer and more time consuming.

Subjects had to perform six runs of the task. The visual or the vibrotactile feedback was provided in alternative runs. Type of feedback of the first run was randomized across subjects.

Control commands and navigation trajectories were recorded, and several indices of performance were computed offline: rate of steps in the ideal path (SIP), rate of steps in an acceptable path (SAP), time to complete the 10 room path, and rate of correct answers to the attentional task (key color).

T-test was performed on these indices to compare the effects of visual versus tactile feedback.

#### 4.3.2. Results

Table 3 reports a summary of the performance indices.

The rate of steps within the ideal path was comparable in the two conditions (80.9% versus 83.7%, *p* > 0.05), in line with studies I and II. Considering slightly swinging trajectories around to the ideal path as acceptable, visual feedback allowed higher performance (92.1% versus 89.2%, *p* = 0.004). Nevertheless, the number of keys incorrectly reported is clearly higher during the runs with visual feedback (86.0% versus 97.5%, *p* = 10^−4^). Given the payload set for wrong answer, this yielded a significantly longer time to destination in the same condition (182 seconds versus 131 seconds,*p* = 2 × 10^−4^).

Remarkably, two of the subjects reported appearance of blue and red keys (which were never delivered), only during runs with visual feedback.

#### 4.3.3. Discussion

The tactile feedback modality was used and compared to the visual while subjects were required to perform a visually guided navigation task. We reduced the experimental variables, by setting up a pseudo-BCI control, which retains the typical inaccuracy, delay, and attention requirements of an EEG-based BCI.

If we only consider the ability of subjects to guide the placeholder towards the gates, the accuracy obtained with visual and tactile feedbacks looks comparable. A deeper analysis, showed that with tactile feedback, subjects tend to stay closer to the ideal path, thus pacing on a more straight line. The most notable difference was in the attentive resources that subjects were able to devote to the task. A significantly higher rate of mistakes was made when visual attention was divided between the control and task monitors.

The subjects reported a good level of comfort in the experimental session lasting about 1 hour. Prolonged tests are needed to assess long-term compliance.

## 5. CONCLUSIONS

The importance of feedback in BCI experimentation is unquestionable, both during the training phase, and at a later stage. Visual feedback is most exploited in this field of research. In this experimental series, we tested how well we can convey an appropriate flow of information into vibrotactile stimulation. To this purpose, we developed a hardware system and a set of software programs that were interfaced to a BCI setup. Information from the BCI system was successfully translated into tactile stimuli, exploiting the features of the sensory channel that physiology are best detectable by users.

In the experiments we conducted the vibrotactile feedback was systematically compared to the usual visual feedback. In summary, we found that tactile feedback (i) permits an appropriate training of users to BCI operation; (ii) does not interfere with simultaneous visual stimuli; (iii) may improve performance when the subject's attention is highly loaded by a simultaneous visual task.

Although these observations have to be confirmed on a larger scale of experimentation with more subjects, it is conceivable to assume that the vibrotactile channel can be effective in relieving the visual channel whenever a dynamic environment overloads the visual channel. In fact, as in the last experimental setting, the user of a BCI system in a real-life context should be able to attend the continuous incoming information both from the BCI feedback itself and the task-relevant information, that is, navigation information, unexpected obstacles, and directions which would mostly be mediated by his/her visual sense. This information processing requires at this stage, a very high level of attentional effort and decrease of performance is likely to occur if this sensory load is not divided into different senses. In this regard, future experiments are needed to explore the natural integration between multimodal feedbacks (visual, auditory, and tactile) in oriented tasks executed under BCI control.

Vibrotactile feedback could be of practical use in applications of BCI technology. Not only would it allow a user to receive a private feedback message, that is, not perceivable by people close to him, but it could be packaged into a wearable device and hidden under clothes, thus improving portability of the system.

An intrinsically multisensorial BCI system can be envisaged, that could deliver BCI-specific information back to the user through the sensory channel (visual, auditory, or tactile) which is less engaged in the current BCI controlled task. This feedback could either be equally shared on different channels, or replicated on each of them. Alternatively, an intelligent system could even dynamically direct the stimuli to the least congested sensory channel.

In conclusion, our findings indicate that the vibrotactile channel can function as a valuable feedback modality in a BCI-controlled setting. Its reliability is comparable to the classical visual feedback, and it can improve performance during tasks that need a focused visual attention.

## Figures and Tables

**Table 1:** Mean classification accuracies for 3 sessions (%). HF, VF: vibrotactile and visual feedback, respectively.

	S1	S2	S3	S4	S5	S6	Mean±SD
HF	77	71	56	71	64	67	68±7
VF	80	67	64	70	67	58	68±7

**Table 2:** The average subject performance in the training sessions 2-3 (TS), vibrotactile feedback sessions (HF), and visual feedback sessions (VF). S1–S3 were given vibrotactile feedback in sessions 4–6; conversely S4–S6 were shown visual feedback in sessions 4–6.

	S1	S2	S3	S4	S5	S6	Mean±SD
TS	62	56	65	68	67	50	61±7
HF	79	70	70	68	59	54	67±9
VF	79	65	65	79	64	53	68±10

**Table 3:** Performances of subjects included in study III. SAP: rate of steps in an acceptable path; SIP: rate of steps in the ideal path.

User	Average control (practice) (%)	Visual feedback				Vibrotactile feedback			

		Average control (task)		Time to destin. (mm:ss)	Correct keys (%)	Average control (task)		Time to destin. (mm:ss)	Correct keys (%)
	
		SAP (%)	SIP (%)			SAP (%)	SIP(%)		
S01	90	90	75	2:20	94%	86	79	2:06	100
S02	79	94	87	2:14	91	91	87	2:07	97
S03	80	89	78	2:37	86	85	78	2:08	100
S04	74	91	81	2:50	86	91	86	1:59	100
S05	81	92	84	3:04	73	90	86	2:17	91
S06	66	89	73	3:33	70	85	74	2:33	88
S07	78	92	82	2:31	91	91	87	2:00	100
S08	74	91	78	2:43	83	91	87	2:03	100
S09	84	95	85	2:10	94	91	86	2:03	100
S10	79	93	86	2:36	86	90	85	2:01	100
S11	64	93	79	2:23	94	92	87	2:02	100
S12	73	89	78	2:25	88	87	80	2:09	97
S13	84	92	80	2:46	81	89	85	2:14	94

Avg.	77.7%	92.1%	80.9%	3:02	86.0%	89.2%	83.7%	2:11	97.5%

## Footnotes

In this experiment, subjects were exposed to a situation similar to that experienced while driving a car along a given path and visually attending the route (task), while monitoring the speed of the vehicle (status) so that it does not exceed the limit. The working hypothesis is that when we compare driving performances when the speed information is given (a) by a visual gauge (same sensory modality challenged by the driving task) or (b) by an auditive beep (bimodal sensory input shared onto two sensory modalities), the second case will lead to better results.
